# Improved data linkage in the Extended Cohort for E-health, Environment and DNA (EXCEED) study through an electronic informed consent (eConsent) and recruitment management system.

**DOI:** 10.23889/ijpds.v7i3.2058

**Published:** 2022-08-25

**Authors:** Xueyang Wang, Emma Adams, Catherine Bee, Anna Guyatt, Catherine John, Richard Packer, David Shepherd, Laura Venn, Martin Tobin, Robert Free

**Affiliations:** 1 University of Leicester

## Objectives

The complexities of the informed consent process for participating in cohort-based medical studies are well-recognised, and the pandemic presented specific challenges related to this. Our response in EXCEED was to build and deploy a local secure eConsent system that was simple to use, provided advanced functionality and improved data linkage.

## Approach

The eConsent system is integrated into a web app (https://exceed.org.uk/) which was written in Python using the Django framework. A unique profile provides participant access to elements of the study, including two-way linkage to REDCap-based surveys and internal bespoke pages (for example an occupation questionnaire backed by a well-established classification) and access to consent to take part in sub-studies. This allowed participants to see which items have been completed or they have taken part in. Administrator tools were also built to enable advanced management and search functionality for dealing with participants queries or data quality issues.

## Results

In 2020, backed by the new eConsent system, and driven by the COVID-19 pandemic, EXCEED undertook both a new wave of recruitment, and re-contacted existing participants to encourage them to take part in COVID related research. Profile registration and management of pre-2020 participants was also enabled by importing their contact details and consent data from legacy tools. Approximately 1000 EXCEED participants gave informed consent using the new system, while ~1000 existing participants registered. Facilitated by improved data quality using the eConsent system, we correctly linked 93% of consented participants to primary care health care records. This high level of data linkage enables research on the causes and consequences of COVID-19 infection, studies of the genomics of disease onset and progression and recall studies.

## Conclusion

We developed a novel eConsent system, which as well as providing online participant registration and improved administration of participants has improved data linkage. Furthermore, the success of the approach has led it to be implemented in other studies with similar requirements.

